# Supplementary material for: Combinatorial optimization of mRNA structure, stability, and translation for RNA-based therapeutics
Source: Nat Commun. 2022 Mar 22;13:1536. doi: 10.1038/s41467-022-28776-w (PMC8940940; doi:10.1038/s41467-022-28776-w)
Supplement: Supplementary file 1 — Supplementary Information [file 41467_2022_28776_MOESM1_ESM.pdf]

SUPPLEMENTARY INFORMATION

Combinatorial optimization of mRNA structure, stability, and translation for RNA-based therapeutics

Kathrin Leppek[1]*, Gun Woo Byeon[1]*, Wipapat Kladwang[2]*, Hannah K. Wayment-Steele[3]*, Craig H. Kerr[1]*, Adele F. Xu[1]**, Do Soon Kim[2]**, Ved V. Topkar[4], Christian Choe[5], Daphna Rothschild[1], Gerald C. Tiu[1], Roger Wellington-Oguri[6], Kotaro Fujii[1], Eesha Sharma[2], Andrew M. Watkins[2], John J. Nicol[6], Jonathan Romano[6,7], Bojan Tunguz[2,8], Fernando Diaz[9], Hui Cai[9], Pengbo Guo[9], Jiewei Wu[9], Fanyu Meng[9], Shuai Shi[9], Eterna Participants[6], Philip R. Dormitzer[9.10], Alicia Solórzano[9], Maria Barna[1‡], Rhiju Das[2‡]

[1] Department of Genetics, Stanford University, Stanford, California 94305, USA
[2] Department of Biochemistry, Stanford University, California 94305, USA
[3] Department of Chemistry, Stanford University, Stanford, California 94305, USA
[4] Program in Biophysics, Stanford University, Stanford, California 94305, USA
[5] Department of Bioengineering, Stanford University, Stanford, California 94305, USA
[6] Eterna Massive Open Laboratory
[7] Department of Computer Science and Engineering, State University of New York at Buffalo, Buffalo, New York, 14260, USA
[8] NVIDIA Corporation, 2788 San Tomas Expy, Santa Clara, CA 95051
[9] Pfizer Vaccine Research and Development, Pearl River, NY, USA
[10] current address: GlaxoSmithKline, 1000 Winter St., Waltham, MA 02453

* these authors contributed equally
‡ co-corresponding authors
Contact: Maria Barna: mbarna@stanford.edu; Rhiju Das: rhiju@stanford.edu

This document includes:

Supplementary Note 1

Supplementary Figures 1 to 13

SUPPLEMENTARY Note 1

In-cell 5' UTR sequence selection to determine rules of efficient translation initiation

Beyond comparing full UTR and CDS regions, we further sought to select for an optimally translating 5' UTR sequence in an unbiased fashion from a complex sequence library (**Supplementary Fig. 2**). Similar to sequence selection by enrichment through direct binding[90] previously performed for mRNA-stabilizing 3' UTR sequences[19], we selected for highly translating transcripts by transfecting an mRNA reporter library with varying 5' UTR sequences and harvesting mRNAs associated with heavy polysomes (**Supplementary Fig. 2a**). We further enriched these libraries for highly translating transcripts over a total of five rounds of selection and re-transfection of the heavily ribosome loaded mRNAs from two independent starting pools (**Supplementary Fig. 2a, b**). We chose to perform two independent replicates at each step because the library complexity is greater than the sequencing depth. We compared them to input sequences of the initial and fifth selection round by RNA-seq. Using the *hHBB* 5' UTR as our baseline (**Supplementary Fig. 2a**), our 5' UTR library design used the first 29 nt of the *hHBB* 5' UTR followed by a 35 nt stretch of random sequences (N35, N=A,C,T,G), which was generated using degenerate oligonucleotide primers, and the consensus Kozak sequence (GCCACCAUGG)[91,92] upstream of the Nluc ORF for a total 70 nt-long 5' UTR.

First, we asked whether 5' UTRs selected to be polysome-associated would increase the protein output compared to *hHBB*. We chose candidate 5' UTRs in which we observed high read counts in the final round (≥15 reads), increasing representation across all selection rounds (FDR≤0.1), and >2-fold enrichment in the last round of selection compared to its input (**Supplementary Fig. 2c**) and performed luciferase reporter assays with these mRNAs (**Supplementary Fig. 2d**). Beside a wide range of luciferase activity driven by candidate 5' UTR mRNAs, we surprisingly observed that none demonstrated luciferase activity that was significantly higher compared to *hHBB* 5' UTR. Thus, although we are selecting for 5' UTR reporter mRNAs of highest ribosome load, this unexpectedly decreases total protein output, which suggests the selected 5' UTRs may have also impacted mRNA stability or translation elongation kinetics; a similar tradeoff is reported in the PERSIST-seq measurements described in the main text (**Fig. 2**).

To determine common features among the selected 5' UTR sequences, we calculated position-specific short k-mer enrichment across the N35 region using *k*pLogo[93] (**Supplementary Fig. 2e**). We observed stronger enrichment/depletion of specific k-mers (165,611 k-mers tested in total) towards the 5' and 3' ends of the N35 stretch (**Supplementary Fig. 2e**). In a confirmatory observation expected from the ribosome scanning model of translation initiation, AUG triplets are

significantly depleted across the N35 region (**Supplementary Fig. 2f**). This effect is periodic and specific to two out-of-frame (frames 1 and 2) AUGs while in-frame AUG (frame 0) is not strongly affected, suggesting the negative impact of the competing upstream start codon except when it is in-frame to result in an N-terminally extended ORF and protein product. A variety of other interesting motifs are further observed, such as the depletion of guanine repeats (for example depletion of GGGG or GGG at the 3' end of the N35, close to the fixed Kozak consensus) and uridine repeats throughout the 5' UTR and enrichment of specific k-mers that suggest formation of short stem-loop structures promoting translation (**Supplementary Data 3**). The latter is especially striking: for example, the 6-mer GUGAAC is strongly enriched towards the 5' positions of the variable N35-mer region; GUGAAC is reverse complement to the last 6 nucleotides of the fixed HBB-29 region (GUUCAC), which would therefore be able to perfectly base-pair with each other and comprises an inverted repeat (**Supplementary Fig. 1g**). The enrichment of the 6-mer peaks at the 4th to 6th nucleotide position downstream of the HBB-29 region, thus favoring an intervening length of 3 nt that would allow a 3-nt loop to form after base-pairing with the 6-mer stretches. Examining other possible inverted repeat k-mers in the variable region as 6-mer reverse complements sliding along the fixed region, we find that the stem may be formed up to around position -30 to the AUG. Such a pattern indicates that folding of a small stem-loop in the middle of the 5' UTR under selection may actually be favored in mRNAs with heavy polysome load. This finding is in contrast to the typical expectations for secondary structures in 5' UTRs to generally repress translation initiation. This finding is interesting because some synthetic small 5' UTR RNA hairpins have previously been found to improve protein expression[94]. In sum, our sequence selection strategy formalizes previously predicted rules for 5' UTR sequences that optimize ribosome load, and motivates an integrated approach to optimization of protein expression that jointly leverages our ribosome load dataset (**Fig. 1**) in parallel with our study of in-cell mRNA stability (**Fig. 2**).

SUPPLEMENTARY FIGURES 1 to 13

Suppl. Figure 1

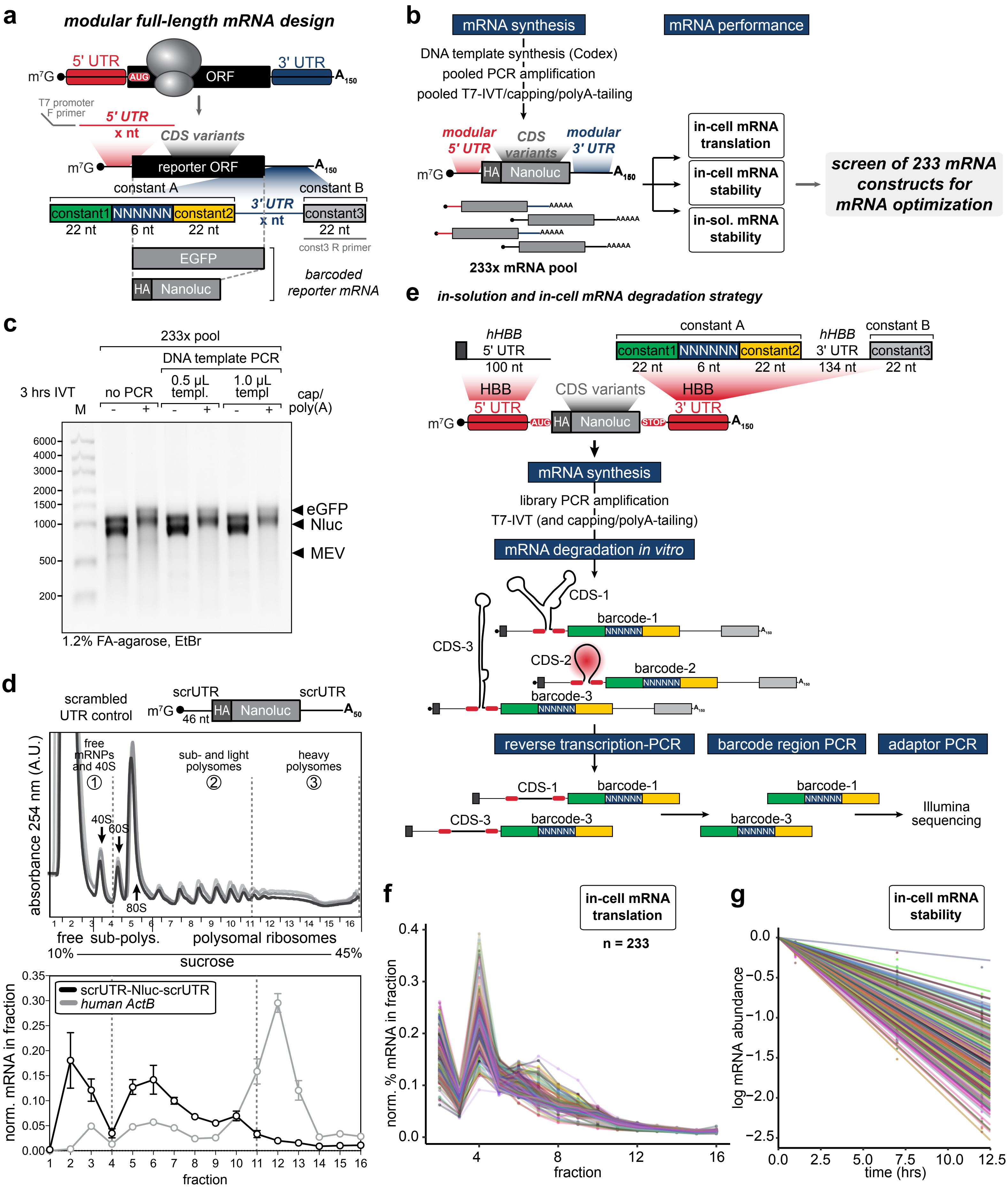

Supplementary Figure 1. mRNA reporter design and in-cell and in-solution workflows with in-cell polysome validation.

(**a**) Schematic for the 3' UTR-barcoded mRNA reporter used to screen mRNA performance in a pooled format. The constant regions and barcode, which flank a variable 3' UTR, were instrumental for amplifying and identifying hundreds of constructs simultaneously in each of the pooled experiments that comprise PERSIST-seq. The DNA templates for full-length mRNAs were synthesized on the Codex platform and amplified in a pooled PCR using primers complementary to the constant region (T7 promoter) preceding the variable 5' UTR, and to the 'constant3' region following the variable 3' UTR.

(**b**) Summary of the workflow to progress from the individually synthesized DNA templates to the *in vitro* synthesized mRNA pool of 233 different constructs. We then use the same mRNA pool to screen mRNA performance in a three-pronged set of in-cell and in-solution expression and stability analyses.

(**c**) Quality control of the 233-mRNA pool on a 1.2% formaldehyde (FA) gel stained with ethidium bromide (EtBr) after 3 hrs of *in vitro* transcription (IVT). The mRNA pool was analyzed before and after capping and polyadenylation. Pooled IVT is equally efficient with the starting template DNA pool with or without PCR-amplification of the DNA template pool. The three major bands corresponding to the three CDS types are indicated. The RiboRuler High Range RNA ladder (Thermo Fisher) is loaded for reference. This result has been repeated independently 3 times with similar results.

(**d**) Polysome fractionation analysis of a transfected mRNA reporter. As an example, the distribution of an mRNA with short scrambled 5' and 3' UTRs 6 hours after transfection into HEK293T cells was compared to the distribution of endogenous human *ActB* mRNA. RNA was extracted from fractions and quantified by qPCR with an RNA spike-in for normalization. Values are plotted as mRNA normalized per fraction. Normalized mRNA in fraction ± SD, n = 3 biologically independent samples.

(**e**) In-solution RNA degradation strategy of barcoded mRNAs containing CDS variants with hHBB 5' and 3' UTRs. The differential degradation of CDS variants depends on their individual CDS structures. mRNA pools are degraded in solution by nucleophilic attack (red circle). After degradation, RT-PCR is performed to selectively amplify mRNAs that remain intact along their full length. Then, the barcode regions of these full-length mRNAs are PCR-amplified, adaptor-ligated, and prepared for Illumina sequencing.

(**f**) Relative normalized abundance of the mRNAs in the 233x library across fractions after sucrose gradient fractionation and RNA sequencing. Normalization and % mRNA per construct and fractions eliminates any construct-specific RT bias.

(**g**) Relative normalized log mRNA abundance of the mRNAs in the 233x library across time points after transfection. Decay curves were fitted assuming a first degree degradation rate. Normalization and log mRNA per construct and time point eliminates any construct-specific RT bias.

Suppl. Figure 2

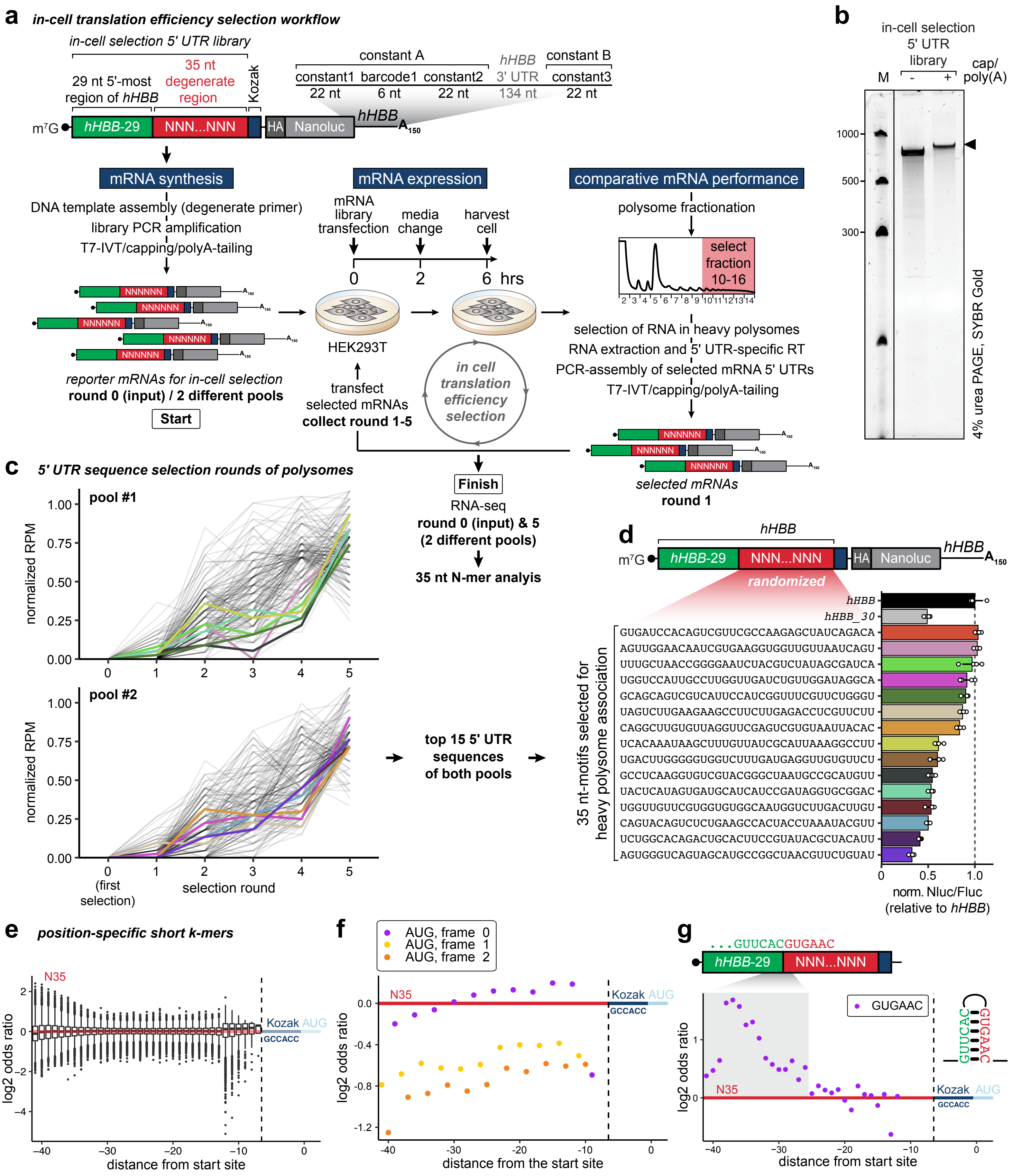

Supplementary Figure 2. Sequential selection of high ribosome loaded mRNAs uncovers 5' UTR sequences that contribute to protein abundance.

(**a**) Overview of the in-cell selection assay designed to uncover 5' UTR sequences that contribute to translational efficiency. First 29 nt of the human HBB 5' UTR was chosen as the fixed 5' region followed by the 35 nt long degenerate region and a constant Kozak consensus sequence (GCCACC). Selection of the variable 35 nt region is introduced by subsequent re-transfection of only the mRNAs purified from the heavy polysomal fractions.

(**b**) Denaturing urea-polyacrylamide gel for the quality control of the in vitro transcribed mRNA 5' UTR selection library before and after the 5' cap and polyA-tail, shown for the selection round 0. All consecutive selection rounds yielded similar libraries. The Low Range ssRNA Ladder (NEB) was loaded for reference. The gel was stained with SYBR Gold (Thermo Fisher). This result has been repeated independently >3 times with similar results.

(**c**) Normalized reads per million (RPM) of the top 5' UTR sequences (FDR≤0.1) over the course of the selection rounds. Colored lines indicate mRNAs that were chosen for luciferase reporter assays (15 in total from two independent starting pools; ≥15 final round read count, ≥2-fold final round enrichment over input).

(**d**) Normalized Nluc/Fluc luciferase activities of the top 15 mRNAs from (c). The 35-nt variable region in the 5' UTR of the polysome selected mRNAs are listed along the y-axis. Their luciferase activity is plotted on the x-axis relative to hHBB. HBB-29 contains only the first 29 nt of the hHBB 5' UTR. Bars indicate the geometric mean of Nluc/Fluc reporter activity ratios normalized to hHBB UTR. Error bars indicate geometric standard deviation. n = 4 biologically independent samples.

(**e**) Boxplot of log2 odds ratios of k-mers (2≤k≤6) between the final polysome selection round and the initial starting pool. Box hinges: 25% quantile, median, 75% quantile, respectively, from bottom to top. Whiskers: lower or upper hinge ±1.5 x interquartile range. Higher variations are observed towards either 5'/3' ends of the 35 nt variable region. Most of the significant k-mers in the 3' positions are depletions.

(**f**) Depletion of out-of-frame (+1- and +2-frames) AUGs within the 35 nt variable region following the polysome selection rounds. In-frame AUGs (0-frame) are weakly depleted or even show minor enrichment closer to the 3' end.

(**g**) Enrichment of the 6-mer motif GUGAAC following polysome selection. GUGAAC is reverse complementary to the 3' end of the fixed 29-nt region of the 5' UTR (GUUCAC). The enrichment towards the 5' end of the variable region and its peak at the 4th to 6th nucleotides downstream of the end of the fixed region may indicate favorability of small stem loop structure for increased ribosome loading.

Suppl. Figure 3

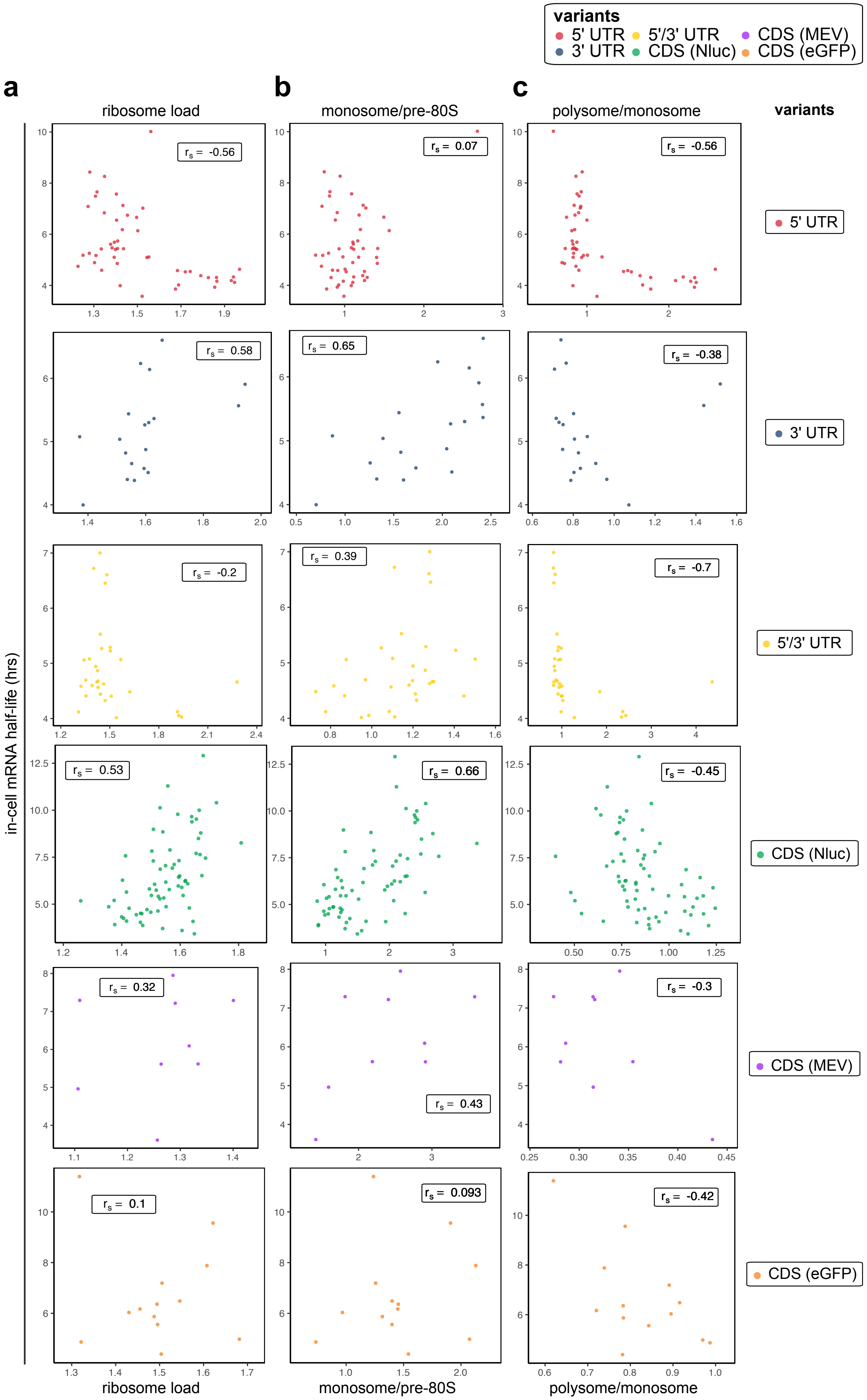

Supplementary Figure 3. Correlations of ribosome load, monosome/pre-polysome and polysome/monosome with in-cell mRNA half-life.

Correlation between in-cell half-life and mean ribosome load across the entire polysome profile (**a**), monosome-to-free 80S subunit ratio (**b**), or polysome-to-monosome ratio (**c**) in HEK293T cells for individual variant groups. Corresponds to **Fig. 2b**.

Suppl. Figure 4

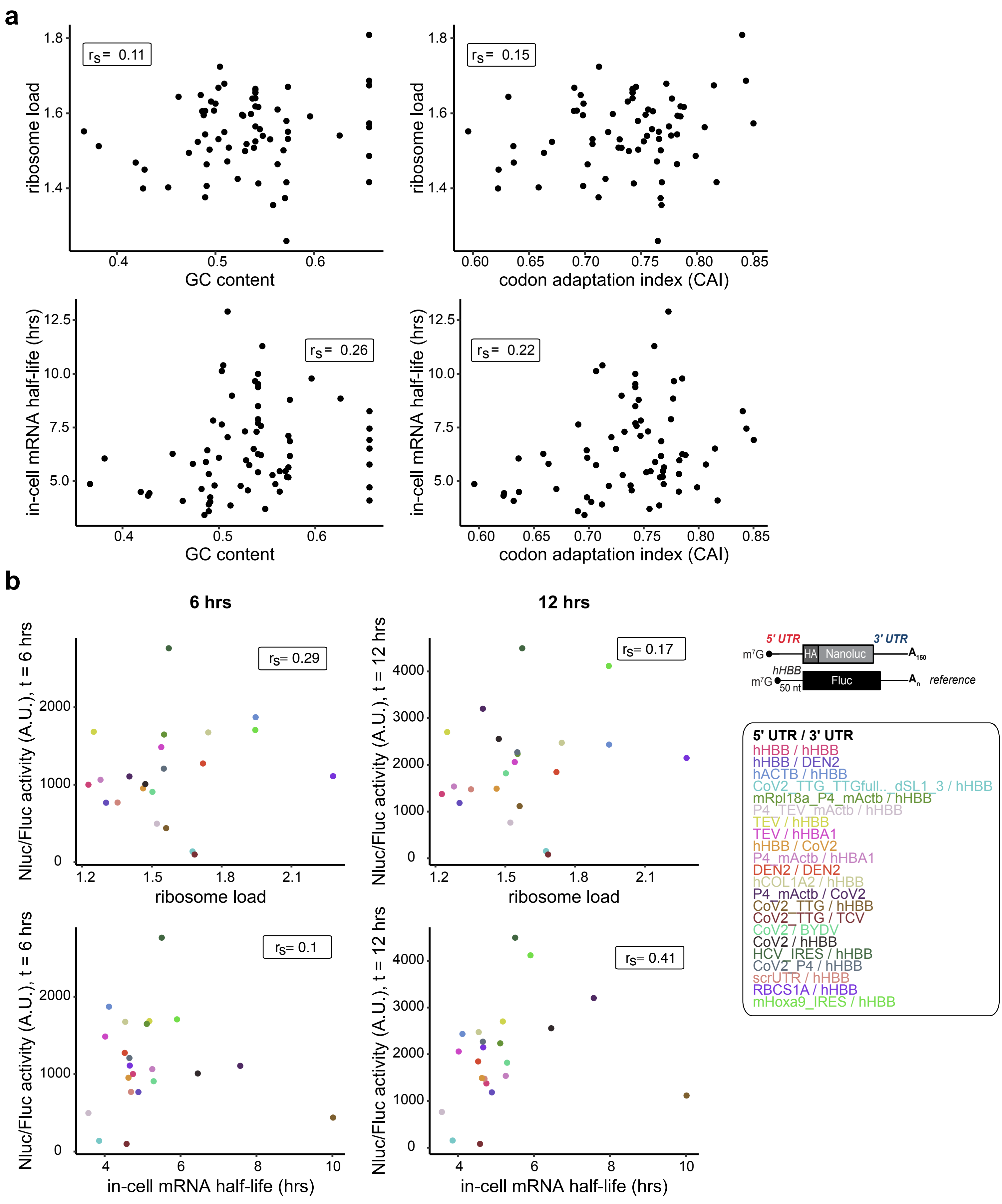

Supplementary Figure 4. Correlations of ribosome load and in-cell mRNA half-life with luciferase expression.

(**a**) CAI and GC correlations with ribosome loads of Nluc CDS variants.
(**b**) Correlation of luciferase with half-life or ribosome loads at each time points.

Suppl. Figure 5

a

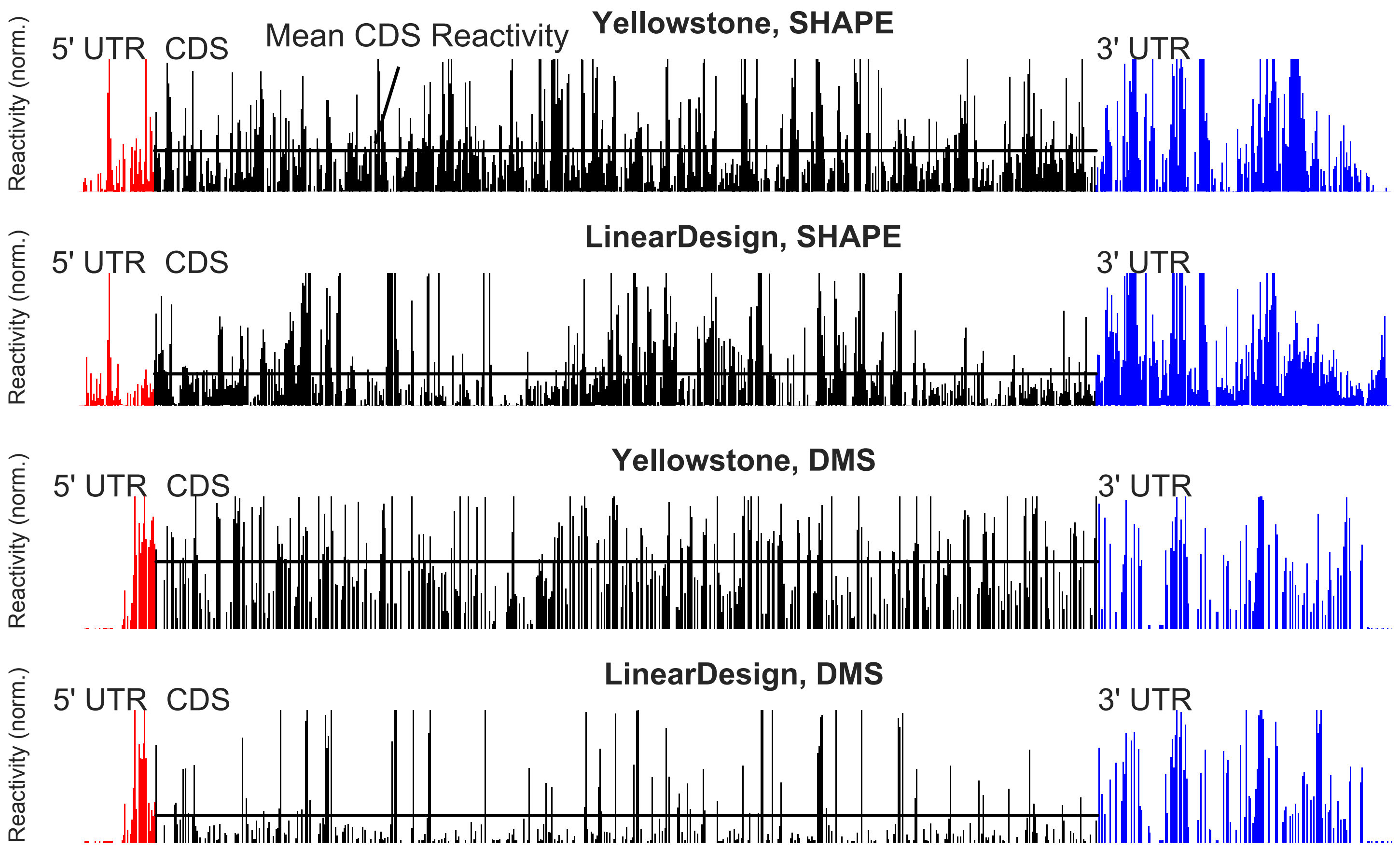

b

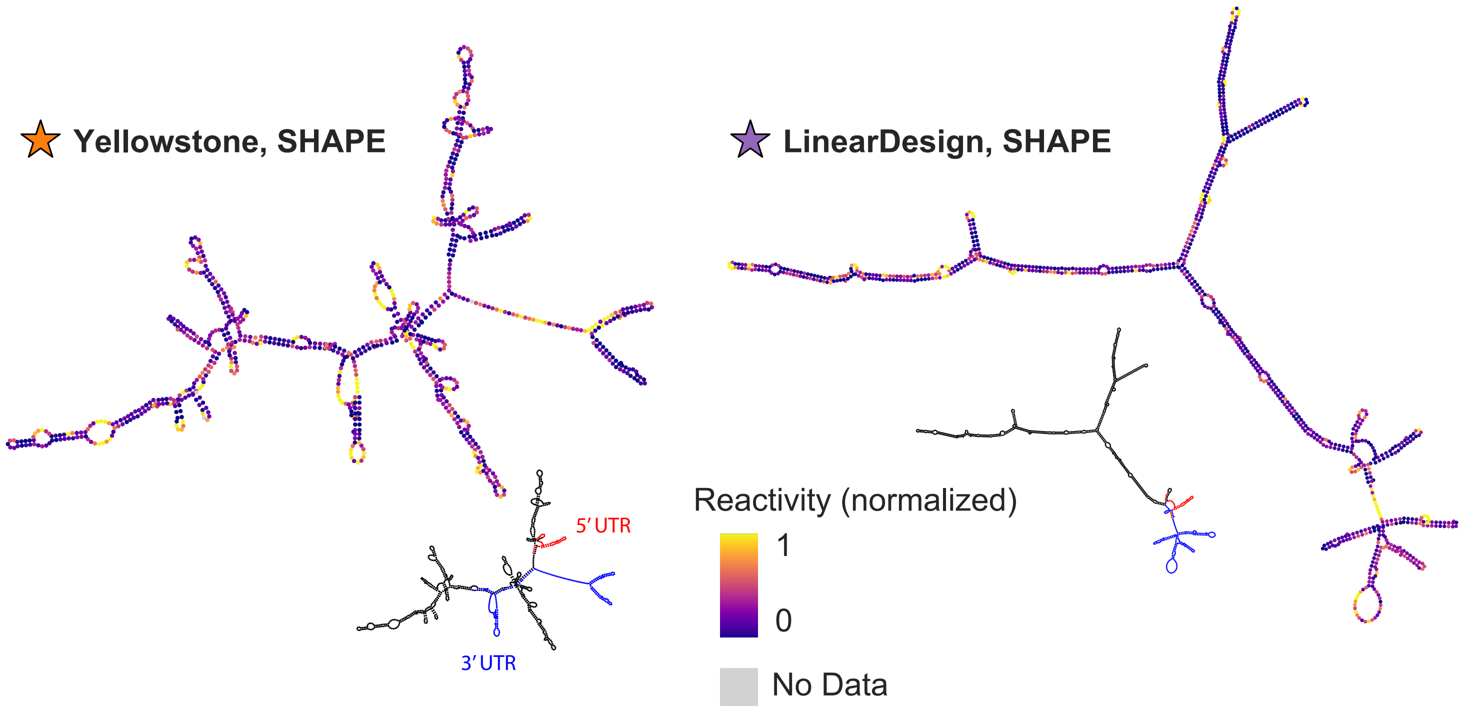

Supplementary Figure 5. Chemical structure probing of Yellowstone and LinearDesign-1 RNAs.

(**a**) SHAPE and DMS reactivity per sequence position of Yellowstone and LinearDesign-1.
(**b**) MFE structures derived using SHAPE reactivity.

Suppl. Figure 6

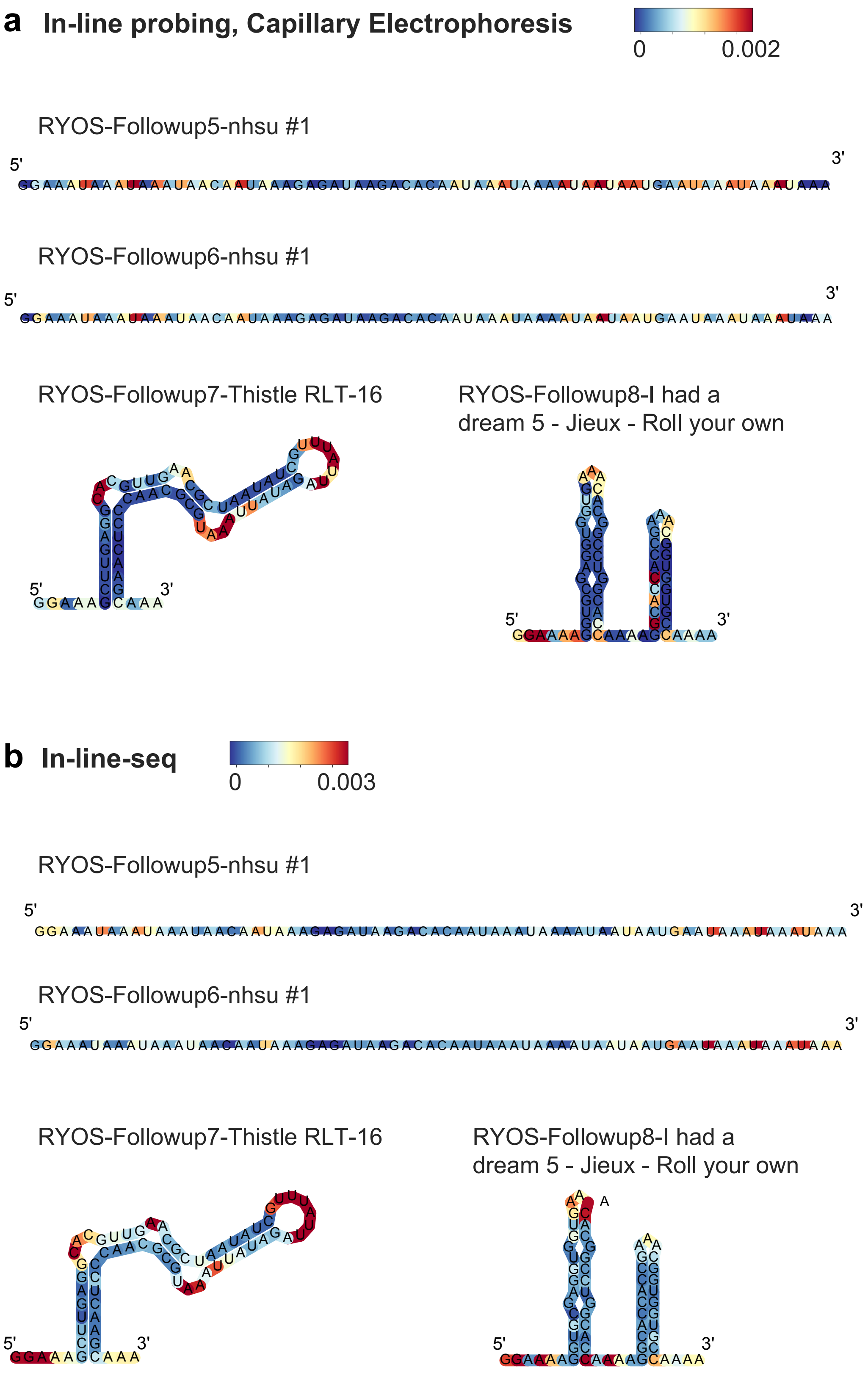

Supplementary Figure 6. Side-by-side comparison of RNA in-line degradation.

Side-by-side comparison of RNA in-line degradation from (**a**) capillary electrophoresis and (**b**) In-line-seq. Coloring was normalized between the 5th and 95th percentile for both data types. Structure is the predicted MFE structure from ViennaRNA.

Suppl. Figure 7

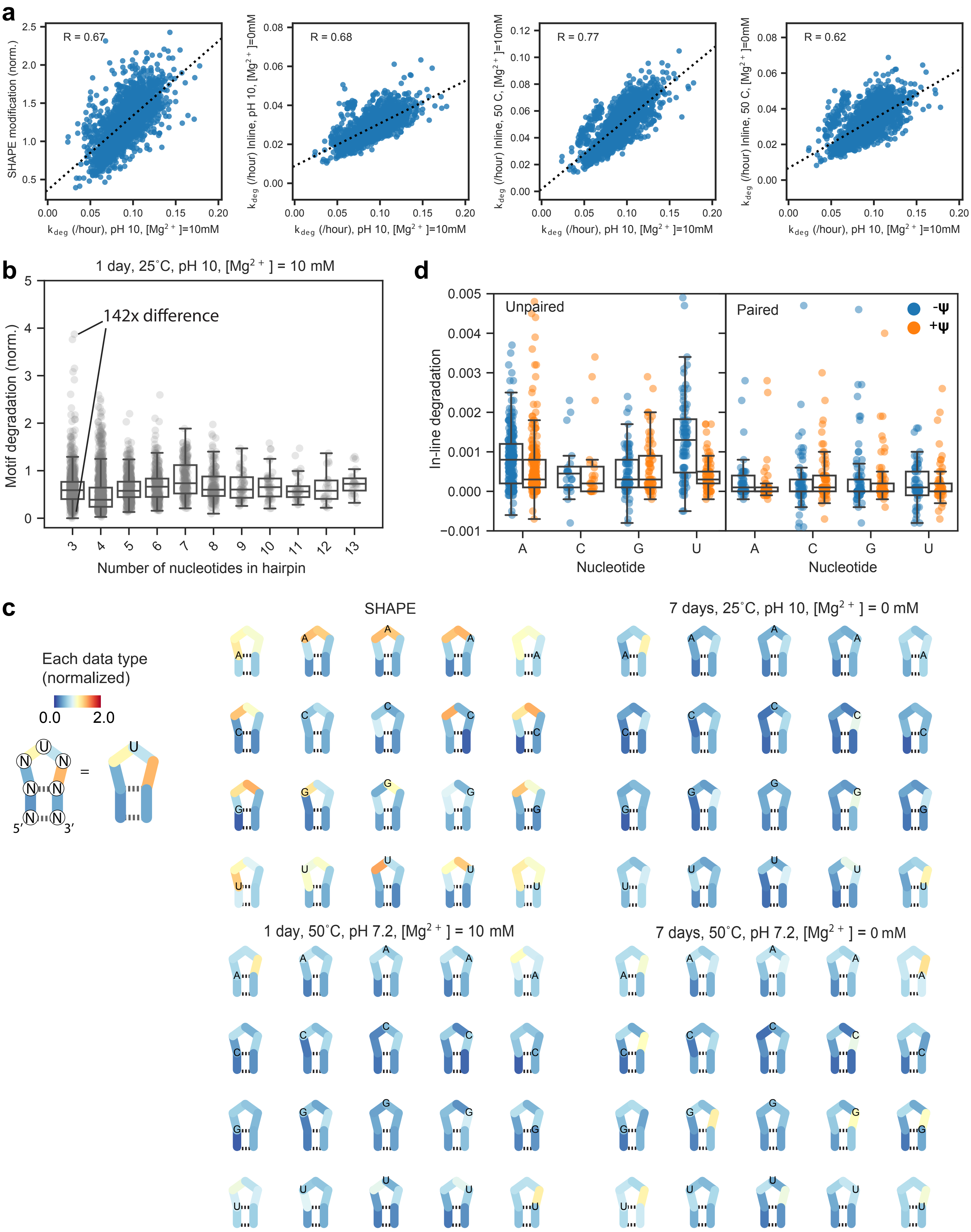

Supplementary Figure 7. Features of RNA degradation as determined by In-line-seq.

(**a**) Correlation of in-line degradation rates per construct at pH 10, 25˚C, [Mg^{2+}] = 10 mM, 1 day conditions, to SHAPE reactivity and other in-line degradation conditions tested.
(**b**) Dynamic range of average reactivity for hairpin loop degradation for in-line degradation at pH 10, 25˚C, [Mg^{2+}] = 10 mM, 1 day conditions. Normalized motif degradation ± SD, n = 1 biologically independent sample. Box hinges: 25% quantile, median, 75% quantile, respectively, from left to right. Whiskers: lower or upper hinge ±1.5 x interquartile range.
(**c**) Sequence/location dependency of triloop reactivity and degradation for other three experimental in-line degradation conditions tested (see **Fig. 3c**).
(**d**) In-line degradation for 8 constructs measured one-by-one with capillary electrophoresis, in absence and presence of pseudouridine. Left panel depicts nucleotides predicted to be unpaired, right panel depicts nucleotides predicted to be paired in ViennaRNA structure. In-line degradation ± SD, n = 1 biologically independent samples. Box hinges: 25% quantile, median, 75% quantile, respectively, from left to right. Whiskers: lower or upper hinge ±1.5 x interquartile range.

Suppl. Figure 8

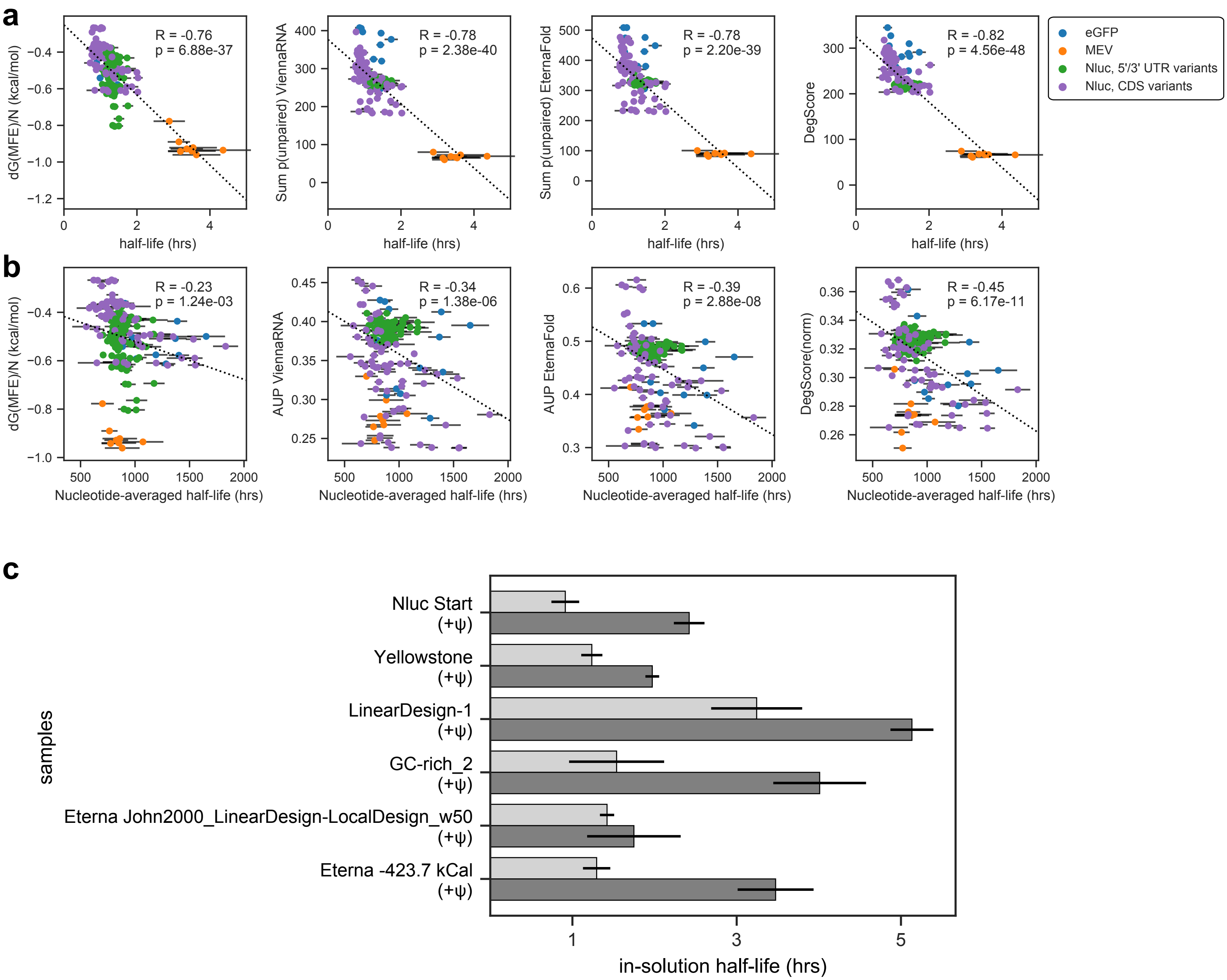

Supplementary Figure 8. Correlation between the 233-mRNA pool in-solution half-life and predictors for RNA degradation.

(**a**) Correlation between in-vitro half-lives and dG(MFE), Sum p(unpaired) calculated in ViennaRNA and EternaFold, and DegScore across all model mRNA types tested. mRNA half-life (hrs) ± SD, n = 3 biologically independent samples. Significance test for Spearman correlation value is two-sided p-value for a hypothesis test whose null hypothesis is that two sets of data are uncorrelated, n = 192. dG(MFE)/N: Free energy of minimum free energy structure divided by length. Sum p(unpaired): Sum of unpaired probability.

(**b**) Correlation between in-vitro half-lives, normalized to RNA length, and dG(MFE), Average p(unpaired) (AUP) in ViennaRNA and EternaFold, and DegScore across the Nanoluciferase and eGFP constructs. Nucleotide averaged half-life (hrs) ± SD, n = 3 biologically independent samples. Significance test for Spearman correlation value is two-sided p-value for a hypothesis test whose null hypothesis is that two sets of data are uncorrelated, n = 192. dG(MFE)/N: Free energy of minimum free energy structure divided by length. AUP: average unpaired probability, i.e. Sum p(unpaired) divided by length.

(**c**) One-by-one characterization of in-vitro half-lives of 6 model mRNAs, characterized with U and with pseudouridine. mRNA half-life data are presented as mean values ± SD, as estimated from one biological experiment via bootstrapped exponential fits as described in Methods.

Suppl. Figure 9

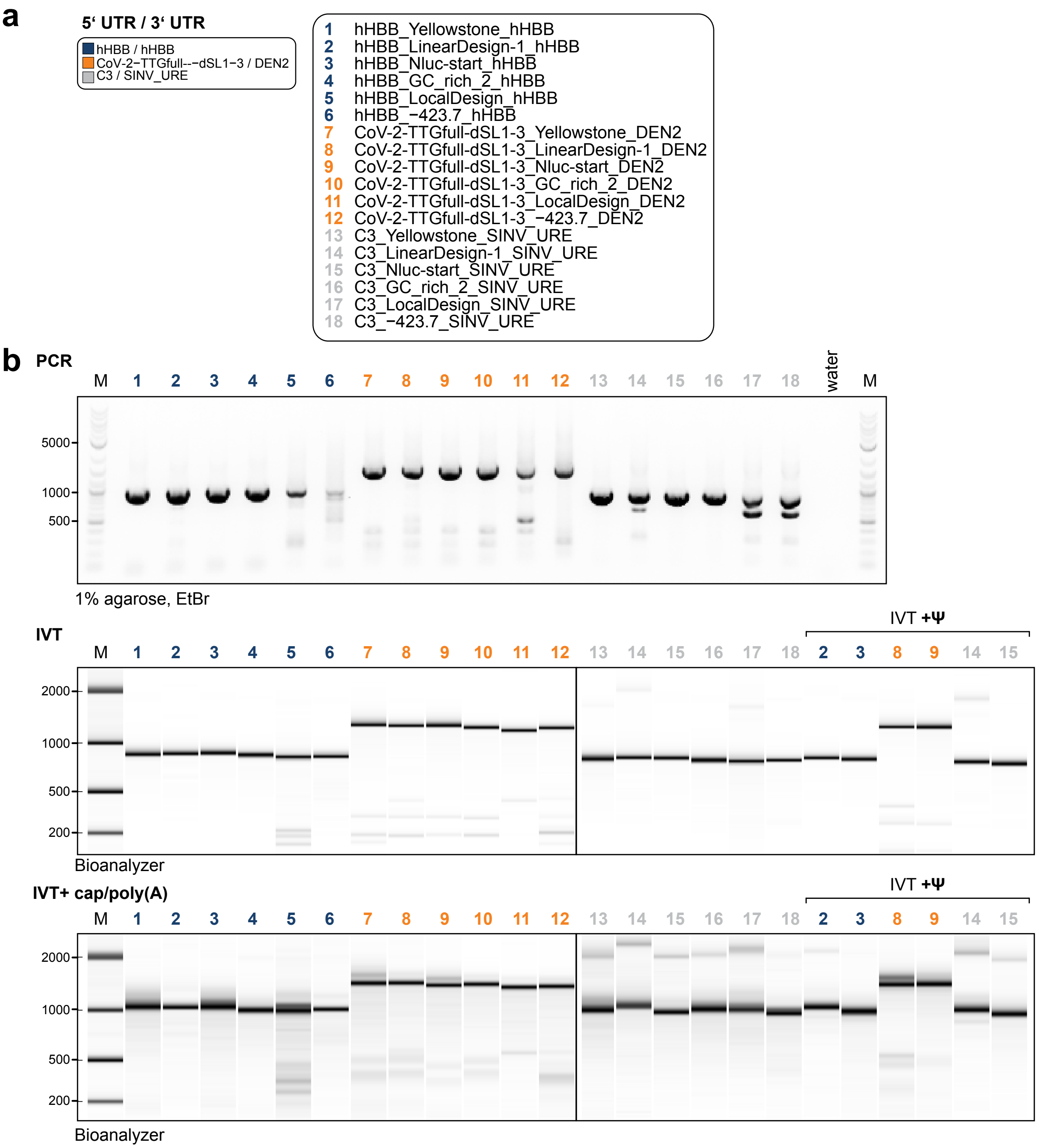

Supplementary Figure 9. Quality control of the workflow of modified RNA synthesis across different RNA designs.

(**a**) mRNAs of different CDS and 5'/3'UTR combinations designed to test their differential impact on protein synthesis. Six CDS constructs were *in vitro* synthesized with different 5' and 3' UTRs. Constructs correspond to **Fig. 4a**.

(**b**) Quality control of the individual constructs after PCR amplification (top), *in vitro* transcription (IVT, middle), and after subsequent cap and polyA-tail modification (bottom), as analyzed by agarose gel electrophoresis and EtBr staining or Bioanalyzer analysis, respectively. Inclusion of ψ in the IVT was tested on six selected constructs. M = molecular weight marker in base-pairs. This result has been repeated independently >3 times with similar results.

Suppl. Figure 10

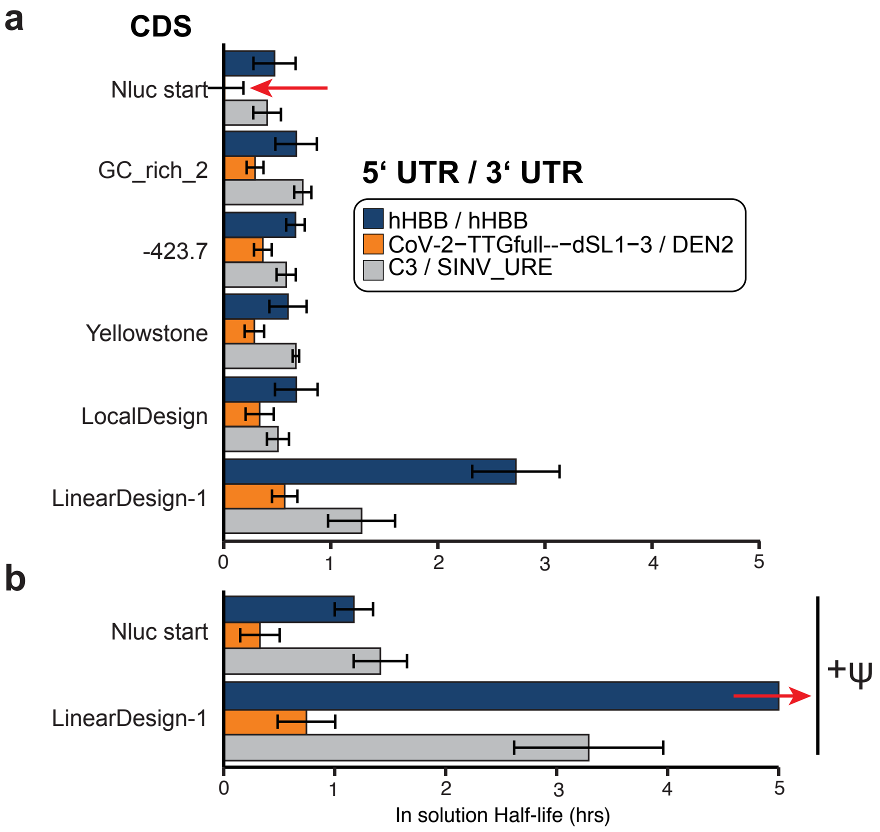

Supplementary Figure 10. Effect of UTR and modified nucleosides on in-solution half-life.

(**a**) 6 select CDS designs were combined with three different pairs of 5' and 3' UTRs and the in-solution half-lives were measured. The half-life of 'Nluc start' with CoV-2-UUG-UUGfull-dSL1-3/DEN2 UTRs (red arrow) could not be accurately measured as it was outside the dynamic range of the experiment; data represent an upper bound. mRNA half-life data are presented as mean values ± SD, as estimated from one biological replicate via bootstrapped exponential fits as described in Methods.

(**b**) Two model RNAs from Panel A were synthesized with pseudouridine and in-solution half-lives were measured. The half-life of "LinearDesign-1" with hHBB/hHBB UTRs containing pseudouridine (red arrow) was not accurately captured as this RNA persisted beyond the range of the experiment; data reflect an approximate upper bound. mRNA half-life data are presented as mean values ± SD, as estimated from one biological replicate via bootstrapped exponential fits as described in Methods.

Suppl. Figure 11

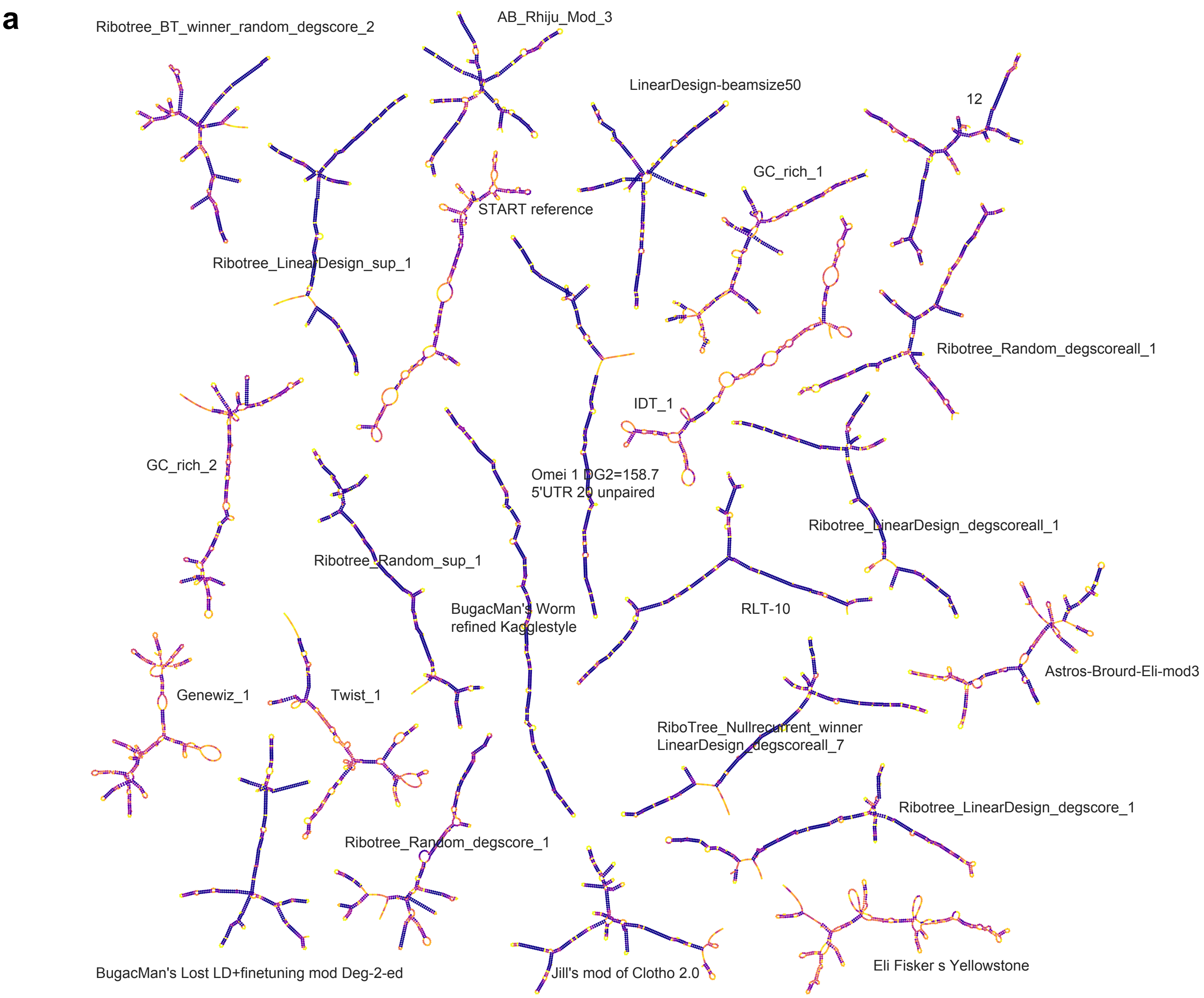

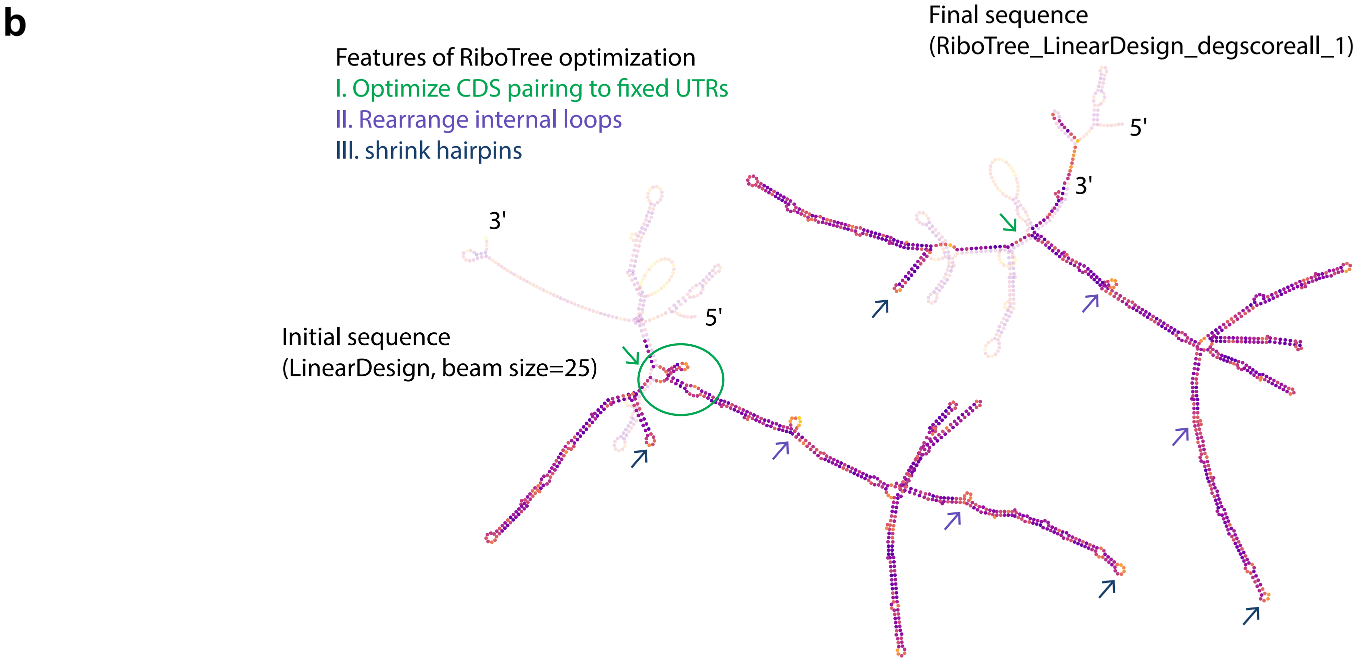

Supplementary Figure 11. Overview of predicted RNA secondary structures of constructs in Fig. 4c.

(**a**) Predicted secondary structures of final tested Nluc constructs, colored by AUP.
(**b**) Comparison of secondary structure of starting sequence used for RiboTree_LinearDesign_degscoreall_1 and annotations of changes.

Suppl. Figure 12

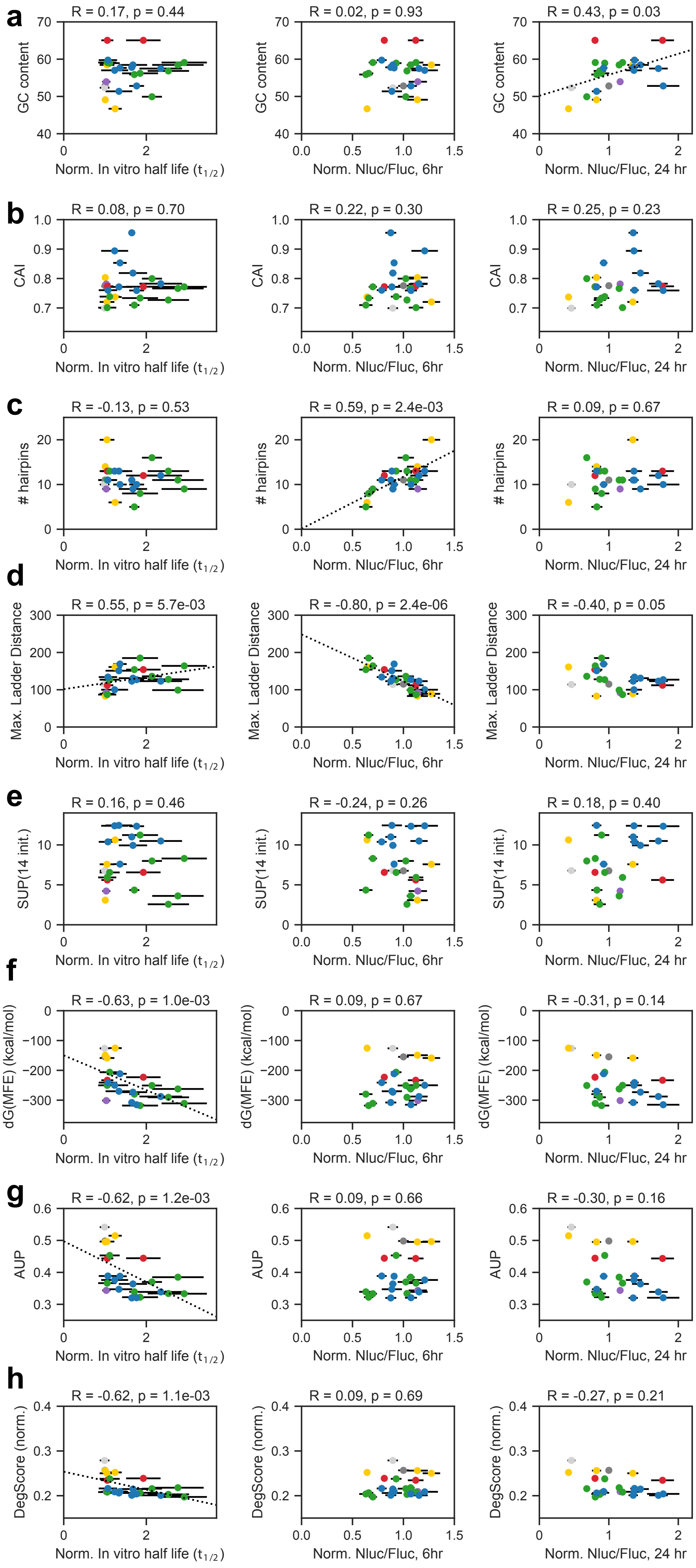

Supplementary Figure 12. Correlation of experimental data for 24 Nluc constructs to predicted degradation and structure metrics.

Correlation of normalized in-solution half-life, normalized Nluc expression at 6 hours and 24 hours, tested for correlation to (**a**) GC content; (**b**) CAI; (**c**) number of hairpins; (**d**) Maximum ladder distance, the maximum length of contiguous helices in the secondary structure; (**e**) SUP(14 init.), the summed unpaired probability of the first 14 nucleotides; (**f**) dG(MFE); (**g**), AUP (average unpaired probability); (**h**) DegScore, not modified to account for degradation suppression due to pseudouridine. Notably, AUP and dG(MFE) have higher correlation to in-solution half-lives than DegScore; this is possibly because the DegScore model was not trained with data on pseudouridine. Significance test for Spearman correlation value is two-sided p-value for a hypothesis test whose null hypothesis is that two sets of data are uncorrelated, n = 24. Error bars indicate standard deviation across n = 3 biologically independent samples for in-solution half-life, n = 4 for normalized Nluc expression at 6 and 24 hours.

Suppl. Figure 13

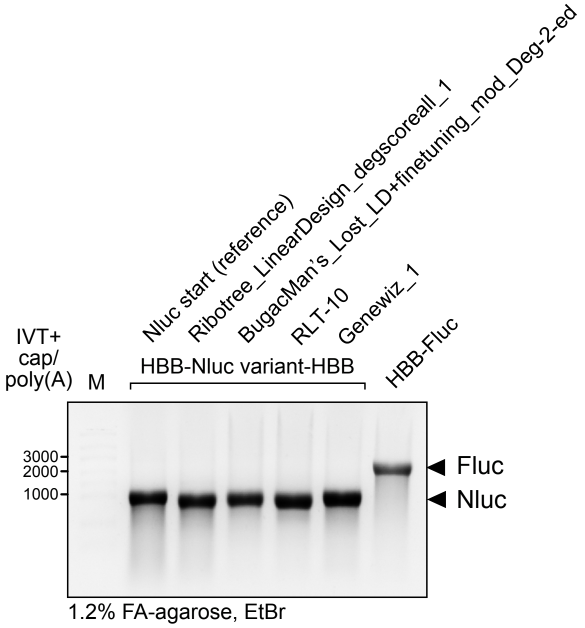

Supplementary Figure 13. Qualitative analysis of Nluc constructs for Polyplex complexation.

Quality control of the mRNA Nluc designs used for Polyplex complexation and mRNA stability and expression analysis. mRNA was analyzed on a 1.2% formaldehyde (FA) gel stained with ethidium bromide (EtBr) after *in vitro* transcription (IVT) and capping and polyadenylation. The RiboRuler High Range RNA ladder (Thermo Fisher) is loaded for reference; molecular weight given in base-pairs. This result has been repeated independently >3 times with similar results.
