# Supplementary material for: Combinatorial optimization of mRNA structure, stability, and translation for RNA-based therapeutics
Source: Nat Commun. 2022 Mar 22;13:1536. doi: 10.1038/s41467-022-28776-w (PMC8940940; doi:10.1038/s41467-022-28776-w)
Supplement: Supplementary file 3 — Description of Additional Supplementary Files [file 41467_2022_28776_MOESM3_ESM.pdf]

Other supplementary material for this manuscript includes the following:

Supplementary Data 1. Attributes for pooled 233 sequences, including nucleotide sequences, sequence annotation, ribosome load, in-solution RNA degradation half-lives, in-cell RNA degradation half-lives, predicted protein expression, and biophysical properties. “NA” indicates values not measured or calculated for select constructs.

Supplementary Data 2. Attributes for CoV-2 5’ UTR mutagenesis sequences

Supplementary Data 3. Frequency and statistics of position-specific k-mers after polysome selection. Observed/expected ratios and binomial test (with normal approximation and Bonferonni correction for multiple testing) p-values are given.

Supplementary Data 4. Normalized DMS and SHAPE (1M7) data on P4-P6, Yellowstone and LinearDesign-1 sequences.

Supplementary Data 5. Significance tests for 24 CDS designs.

Supplementary Data 6. Attributes for 24 CDS designs, including designer/source, nucleotide sequences, in-solution half-lives, luciferase expression values, biophysical properties, and predicted structural metrics.

Supplementary Data 7. List of primers and construct layouts.

Supplementary Data 8. Eterna OpenVaccine participants List of primers and construct layouts

Supplementary Data 9. List of OpenVaccine donors.
