# Supplementary material for: Combinatorial optimization of mRNA structure, stability, and translation for RNA-based therapeutics
Source: Nat Commun. 2022 Mar 22;13:1536. doi: 10.1038/s41467-022-28776-w (PMC8940940; doi:10.1038/s41467-022-28776-w)
Supplement: Supplementary file 5 — Reporting Summary [file 41467_2022_28776_MOESM5_ESM.pdf]

nature research

Corresponding author(s): Maria Barna, Rhiju Das

Last updated by author(s): 4/8/2021

Reporting Summary

Nature Research wishes to improve the reproducibility of the work that we publish. This form provides structure for consistency and transparency in reporting. For further information on Nature Research policies, see our Editorial Policies and the Editorial Policy Checklist.

Statistics

For all statistical analyses, confirm that the following items are present in the figure legend, table legend, main text, or Methods section.

n/a	Confirmed	
☐	☒	The exact sample size (*n*) for each experimental group/condition, given as a discrete number and unit of measurement
☐	☒	A statement on whether measurements were taken from distinct samples or whether the same sample was measured repeatedly
☐	☒	The statistical test(s) used AND whether they are one- or two-sided<br>*Only common tests should be described solely by name; describe more complex techniques in the Methods section.*
☒	☐	A description of all covariates tested
☐	☒	A description of any assumptions or corrections, such as tests of normality and adjustment for multiple comparisons
☐	☒	A full description of the statistical parameters including central tendency (e.g. means) or other basic estimates (e.g. regression coefficient) AND variation (e.g. standard deviation) or associated estimates of uncertainty (e.g. confidence intervals)
☐	☒	For null hypothesis testing, the test statistic (e.g. *F*, *t*, *r*) with confidence intervals, effect sizes, degrees of freedom and *P* value noted<br>*Give P values as exact values whenever suitable.*
☒	☐	For Bayesian analysis, information on the choice of priors and Markov chain Monte Carlo settings
☒	☐	For hierarchical and complex designs, identification of the appropriate level for tests and full reporting of outcomes
☐	☒	Estimates of effect sizes (e.g. Cohen's *d*, Pearson's *r*), indicating how they were calculated

Our web collection on statistics for biologists contains articles on many of the points above.

Software and code

Policy information about availability of computer code

Data collection	All code and software used is described in the methods and github links are given. Scripts for the measurement of in-solution mRNA stability by capillary electrophoresis are available at https://github.com/DasLab/openvaccine-CE-analysis. PERSIST-seq processing pipeline is available at https://github.com/barnalab/persist.<br>cutadapt 2.10, umi_tools 1.0.1, bowtie2 2.4.1, samtools 1.9, UMIcollapse (https://github.com/Daniel-Liu-c0deb0t/UMICollapse), R 4.1.0, errors 0.3.6, limma 3.50.0
Data analysis	All code and software used is described in the methods and github links are given. Code to produce linear DegScore model and degradation prediction calculations is available at https://github.com/eternagame/DegScore.

For manuscripts utilizing custom algorithms or software that are central to the research but not yet described in published literature, software must be made available to editors and reviewers. We strongly encourage code deposition in a community repository (e.g. GitHub). See the Nature Research guidelines for submitting code & software for further information.

Data

Policy information about availability of data

All manuscripts must include a data availability statement. This statement should provide the following information, where applicable:

- Accession codes, unique identifiers, or web links for publicly available datasets
- A list of figures that have associated raw data
- A description of any restrictions on data availability

Raw sequencing data for PERSIST-seq and In-line-seq experiments are deposited in the Gene Expression Omnibus (GEO) under accession number GSE173083.

Single-nucleotide-resolution in-line probing and SHAPE data are deposited at the following RNA Mapping Database 98 (http://rmdb.stanford.edu) under the

following accession numbers.
In-line-seq datasets:
RYOS1_NMD_0000 (no modification)
RYOS1_MGPH_0000 ([Mg2+] = 10 mM, pH = 10, 24˚C, 1 day)
RYOS1_PH10_0000 ([Mg2+] = 0 mM, pH = 10, 24˚C, 7 days)
RYOS1_MG50_0000 ([Mg2+] = 10 mM, pH = 7.2, 50˚C, 1 day)
RYOS1_50C_0000 ([Mg2+] = 0 mM, pH = 7.2, 50˚C, 7 days)
SHAPE_RYOS_0620 (SHAPE 1M7 reactivity)
One-by-one follow ups
RYOSFL_MOD_0001
RYOSFL_MOD_0002
RYOSFL_MOD_0003
RYOSFL_MOD_0004
RYOSFL_MOD_0005
RYOSFL_MOD_0006
RYOSFL_MOD_0007
RYOSFL_MOD_0008

Field-specific reporting

Please select the one below that is the best fit for your research. If you are not sure, read the appropriate sections before making your selection.

☒ Life sciences ☐ Behavioural & social sciences ☐ Ecological, evolutionary & environmental sciences

For a reference copy of the document with all sections, see nature.com/documents/nr-reporting-summary-flat.pdf

Life sciences study design

All studies must disclose on these points even when the disclosure is negative.

Sample size	Sample size was chosen based on the expected potential effect sizes and based on the variabilities typically seen for each type of experiment performed as previously reported in the literature or practically experienced in the field.
Data exclusions	Time courses in which the observed fraction intact exceeded the fitted exponential by more than 0.05 in the last time point signaled RT-PCR amplification of misprimed to generate non-full-length products and were filtered out of downstream analysis.
Replication	Each replicate comprises an independent cell culture, transfection, sample collection and quantitative analysis per condition. The exact number of replicates vary for different experiments but are always clearly stated in the figure panel or figure legend.
Randomization	Randomization is not relevant because condiditons were constructed and there was not subjective allocation of samples to experimental groups.
Blinding	Blinding was not considered in this study as the experiments are not based on subjective measures.

Reporting for specific materials, systems and methods

We require information from authors about some types of materials, experimental systems and methods used in many studies. Here, indicate whether each material, system or method listed is relevant to your study. If you are not sure if a list item applies to your research, read the appropriate section before selecting a response.

Materials & experimental systems

n/a	Involved in the study	
☒	☐	Antibodies
☐	☒	Eukaryotic cell lines
☒	☐	Palaeontology and archaeology
☒	☐	Animals and other organisms
☒	☐	Human research participants
☒	☐	Clinical data
☒	☐	Dual use research of concern

Methods

n/a	Involved in the study	
☒	☐	ChIP-seq
☒	☐	Flow cytometry
☒	☐	MRI-based neuroimaging

Eukaryotic cell lines

Policy information about cell lines

Cell line source(s)	HEK293T (ATCC: CRL-3216)
Authentication	Cell lines are not authenticated. They are obtained from ATCC.
Mycoplasma contamination	Cell lines are not tested for mycoplasma.
Commonly misidentified lines (See ICLAC register)	No commonly misidentified lines were used in this study.
